# Cutaneous and Pulmonary Manifestations: COVID-19 Virus or Coccidioidomycosis?

**DOI:** 10.7759/cureus.15060

**Published:** 2021-05-16

**Authors:** Dania A Shah, Sheridan James, Ijeoma U Uche, Rustan Sharer, Priya Radhakrishnan

**Affiliations:** 1 Internal Medicine, HonorHealth, Scottsdale, USA; 2 Internal Medicine & Academic Affairs, HonorHealth, Scottsdale , USA

**Keywords:** coccidioidomycosis, covid 19, anchoring bias

## Abstract

COVID-19 viral pandemic continues to manifest itself in the form of various clinical symptoms. Due to concerns of COVID-19 in the setting of high rates of false-negative, there is increased likelihood of anchoring bias. We present a case of a 48-year-old white female who presented with two weeks of dry cough and diffuse pruritic nodular cutaneous rash. Patient was exposed to a colleague who tested positive for COVID 19. Initial visits were conducted virtually and workup was negative for COVID-19. Patient was offered supportive care; however, her symptoms continued to worsen. Subsequent workup was positive for left lower lobe nodular opacity on the chest X-ray, follow up CT chest showed demonstrated a focal 3.4 cm infiltrate in the left lower lobe pleural base posteriorly, blood workup was positive for eosinophil count, elevated liver enzymes and positive coccidioides antibody IgG and IgM. This case highlights the importance of avoiding anchoring bias when creating differential diagnoses and triaging patients.

## Introduction

Cutaneous involvement is the most common manifestation of disseminated coccidioidomycosis. There are a wide range of skin presentations such as erythema nodosum, erythema multiforme, etc. [1]. Recognizing coccidioidomycosis early is imperative, particularly during the COVID-19 pandemic which can imitate the symptoms of cocci, leading to anchoring bias. Recent studies have confirmed the association of COVID-19 with cutaneous presentations, along with pulmonary symptoms [2]. Establishing correct diagnosis and proper treatment is the key to avoid further complications which can be life-threatening.

## Case presentation

**Figure 1:**
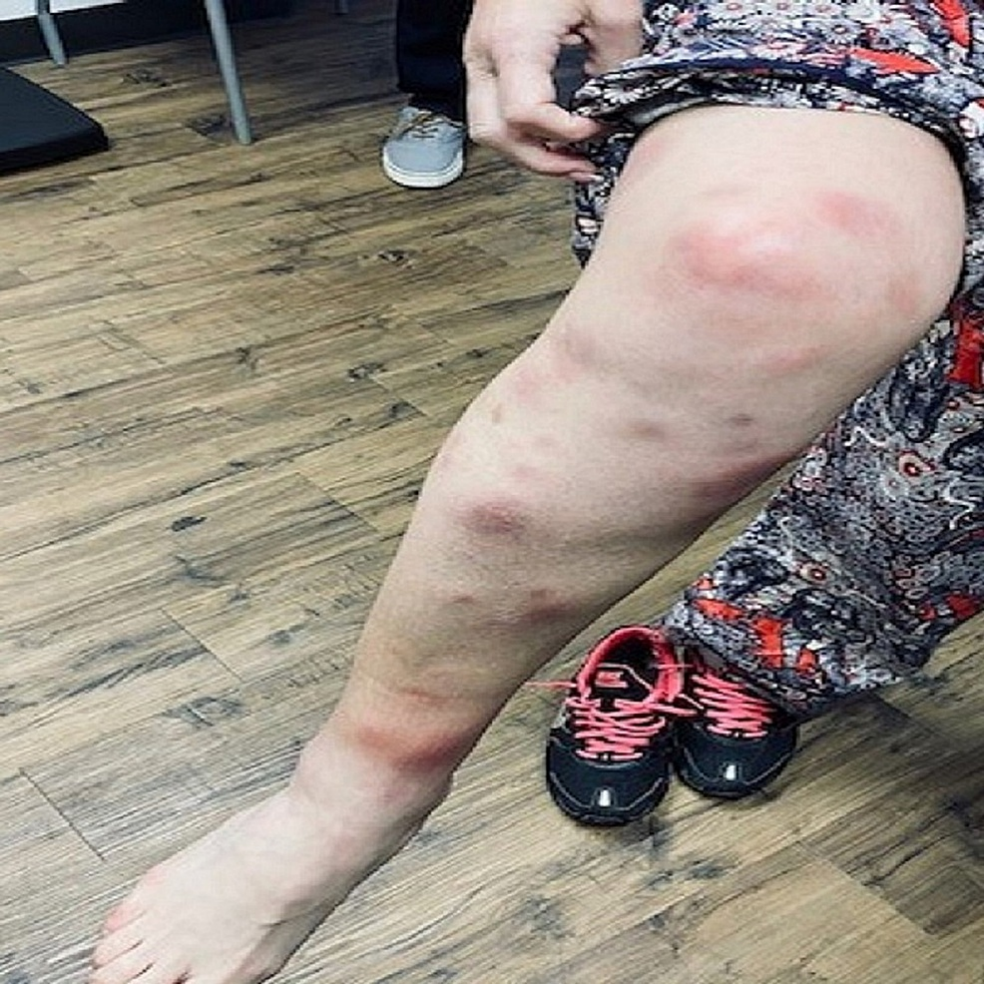
Multiple well-demarcated, raised erythematous cutaneous lesions.

**Figure 2:**
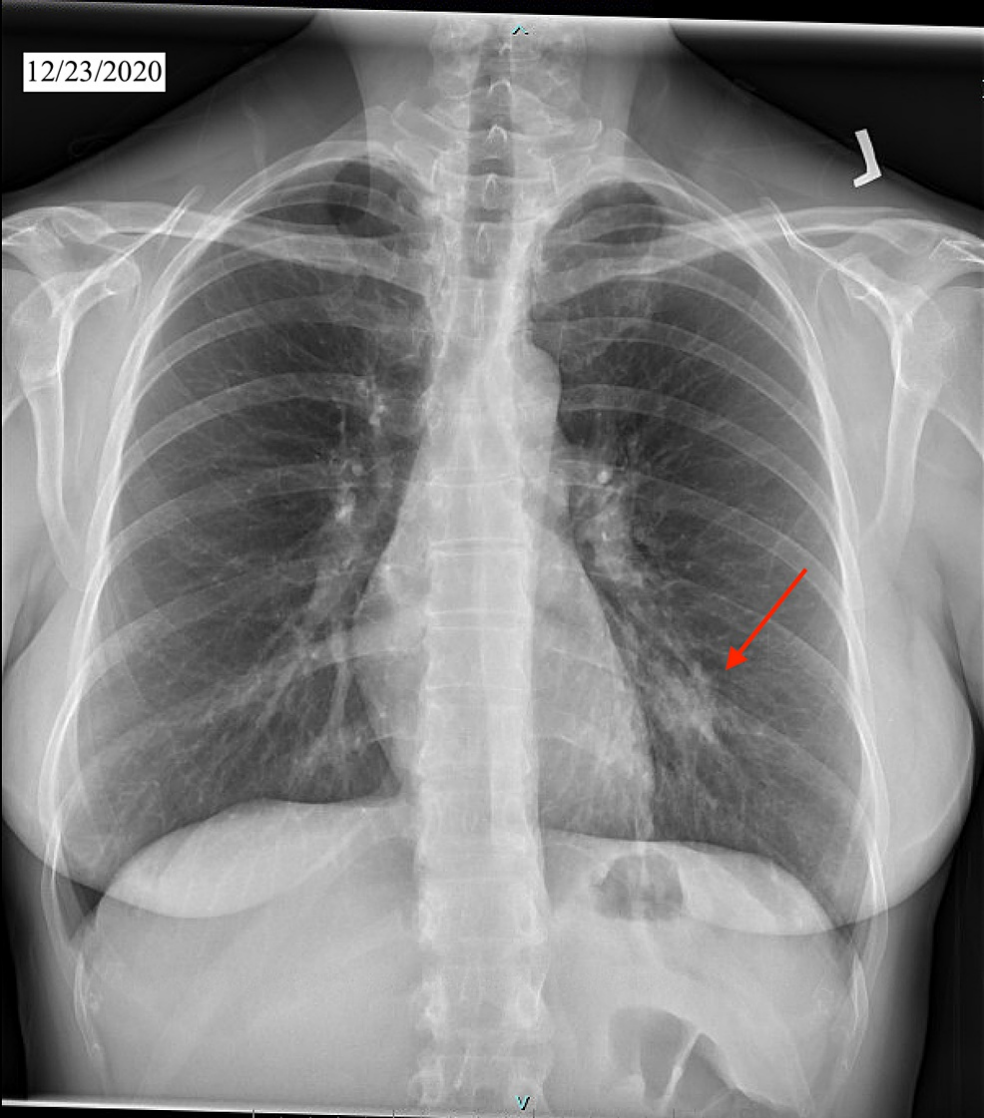
Chest X-ray from 12/23/2020.

**Figure 3:**
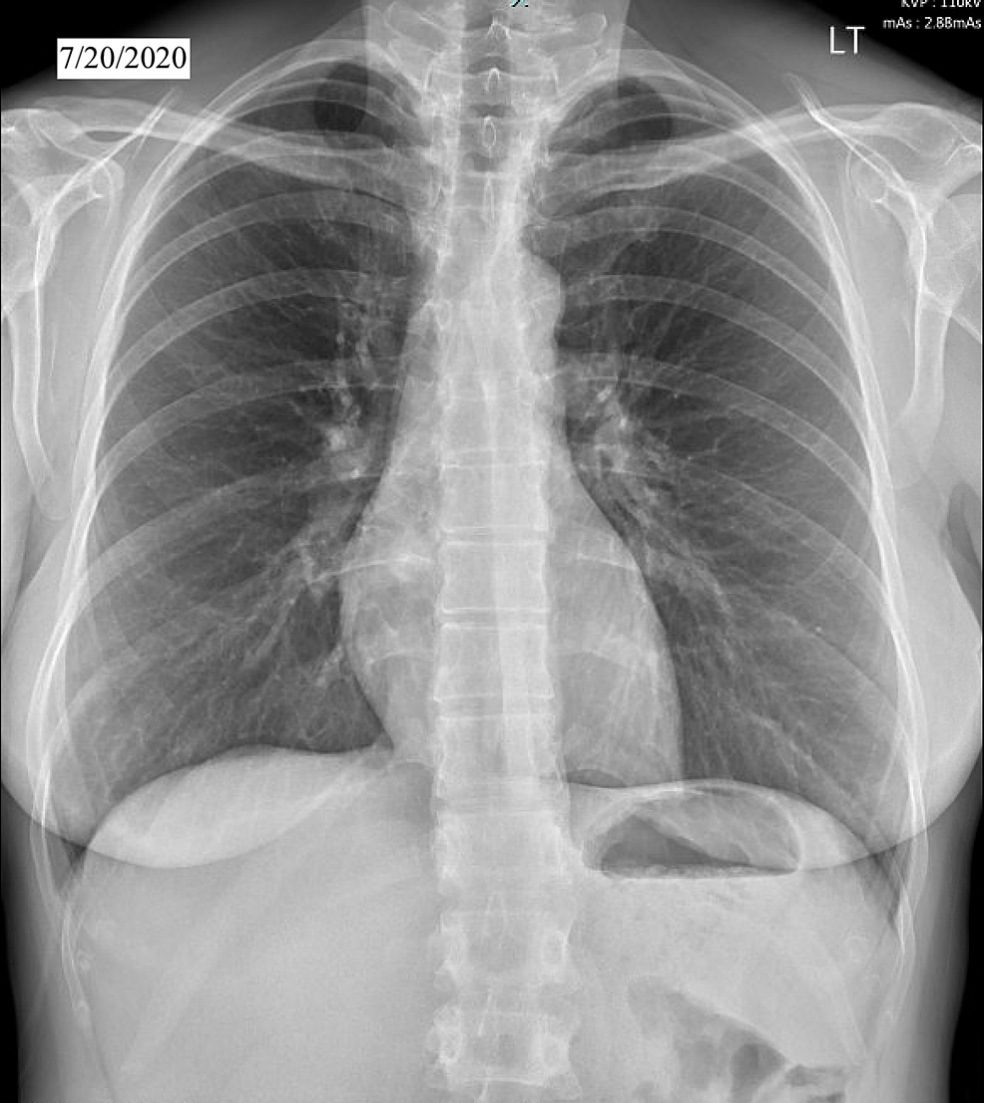
Chest X-ray from 7/20/2020. Normal chest X-ray.

**Figure 4:**
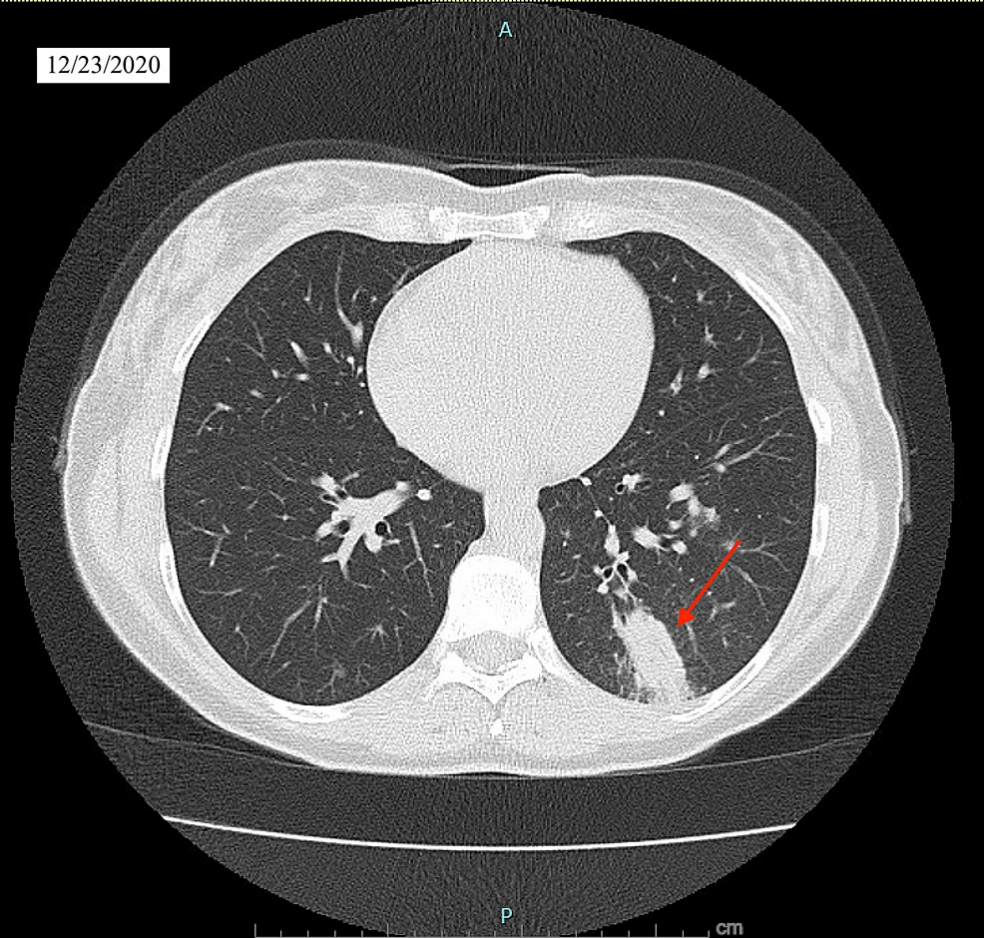
CT chest 12/23/2020.

We present a case of a 48-year-old white female patient with past medical history of smoking, gastroesophageal reflux disease, urinary incontinence, bronchitis who presented with two weeks of dry cough, and a diffuse pruritic nodular cutaneous rash on her back, arms and lower extremities in our outpatient clinic. The patient reported exposure to a colleague who tested positive for COVID-19 virus. Given the heavy burden of COVID-19, she was sent for testing and was recommended quarantine and supportive treatment. Her initial COVID 19 antigen testing was reported as negative. However, given the high false-negative rates of the test and patient’s symptoms consistent with COVID 19, initial management comprised of supportive treatment in the absence of hypoxia. Patient did not improve and continued to experience new cutaneous lesions and worsening respiratory symptoms. Patient remained hemodynamically stable with blood pressure 118/58, HR 87, and SpO2 98% on room air. On physical exam, several well-demarcated raised, tender and erythematous lesions were noted on the back, upper and lower extremities (Figure 1). The rest of the exam was unremarkable. Chest X-ray (CXR) and laboratory tests were ordered. CXR on 12/23/2020 showed new left lower lobe nodular opacity (Figure 2) compared with CXR on 7/20/2020 which was normal (Figure 3). Following the positive CXR finding, CT chest was ordered for a detailed evaluation of the opacity which demonstrated a focal 3.4 cm infiltrate in the left lower lobe pleural base posteriorly while the right lung was unremarkable (Figure 4). Complete blood count was positive for slightly elevated absolute eosinophilic count (Table 1), comprehensive metabolic panel was pertinent for elevated liver enzymes (Table 2) and other pertinent lab finding was positive for serum coccidioides antibody IgG and IgM (Table 3). Other labs were negative for antinuclear antibody, cyclic citrullinated peptide and rheumatoid factor. Hepatitis serologies were negative. Prothrombin time and partial thromboplastin time were slightly elevated. We performed punch biopsy for the proper characterization of one of the skin lesions; pathology reported mild superficial dermal perivascular inflammation, possibly from exaggerated immune response.

**Table 1:** Complete blood count. WBC: white blood cell.

	Reference range	12/31/2020
WBC	4-10.9x10^3^/uL	9.9
Hemoglobin	12.0-16.0 g/dL	14
Platelets	130-450 K/uL	424
Neutrophil Ab	1.48-8.32x10^3^/uL	6.66
Lymphocyte Ab	0.90-3.50x10^3^/uL	1.76
Monocyte Ab	0.26-0.80x10^3^/uL	0.65
Eosinophils Ab	0.00-0.62x10^3^/uL	0.72
Basophils Ab	0.00-0.10x10^3^/uL	0.10

**Table 2:** Trend of liver function test. ALT: alanine aminotransferase; AST: aspartate transaminase.

	Reference range	12/31/20	12/28/20	12/23/20	2/27/20
Alkaline phosphatase	38-126 IU/L	284	194	135	37
ALT	0-54 IU/L	174	145	57	19
AST	0-41 IU/L	94	104	33	18

**Table 3:** Coccidioidomycosis serologies. IgG: immunoglobulin G; IgM: immunoglobulin M.

	Reference range	12/23/2020
Coccidioides IgG, blood	Negative	Positive!
Cocci IgM, blood	Negative	Positive!
Coccidioides Ab, IgG (IMDF)	Negative	Positive!
Coccidioides Ab, IgM (IMDF)	Negative	Positive!
Coccidioides titer	< 1:2	< 1:2

With the initiation and ongoing treatment with Fluconazole, patient showed considerable improvement in her symptoms. Follow up hepatic function panel showed down-trending liver enzymes and repeat serum coccidioides antibody testing was negative. Patient continues to follow up in our clinic as well as with infectious disease.

## Discussion

With a wide spectrum of clinical presentations, it is safe to say that coccidioidomycosis belongs to the group of diseases known as the imitator. Incidence of various types of cutaneous symptoms is one of the frequent reported extrapulmonary manifestations in coccidioidomycosis. Several studies have documented skin findings associated with COVID-19 viral infection, similar to that of cutaneous manifestations seen in patients affected with coccidioidomycosis [2-4]. The pathophysiology involves either a hypersensitivity reaction as part of the immune response, direct inoculation or dermatomal dissemination [5]. The diagnosis of coccidioidomycosis requires high index of clinical suspicion along with blood work consisting of complete blood count, liver function test and coccidioidomycosis serum serologies as well as imaging such as CXR [6]. Punch biopsy findings, in this case, was suggestive of reactive presentation of the skin lesions. This case underscores the importance of anchoring bias which is becoming a common norm in medicine during the COVID-19 pandemic. Anchoring bias is a cognitive bias in which an individual relies too heavily on a specific piece of information in order to make a decision [7]. Another reason for anchoring bias in this particular case can be attributed to the fact that the initial visits of the patients were conducted virtually which made it harder to assess the severity of the skin lesions in detail [8].

Treatment of coccidioidomycosis with antifungal is dose-dependent and the duration is based on the extent of the disseminated symptoms as the chronicity of the coccidioidomycosis infection. In coccidioidomycosis without meningeal symptoms such as in this case, treatment consists of fluconazole for three to six months [9].

## Conclusions

This case emphasizes the importance of keeping a broad differential and avoiding anchoring bias in medicine, especially as COVID-19 virus symptomatology continues to unfold as a mimicker of other diseases. Early recognition of coccidioidomycosis and prompt treatment were crucial to preclude the meningeal dissemination of the disease and to expedite patient’s recovery.
